# Advanced Metastatic Melanoma With C-KIT (CD117) Immunohistochemical Expression: A Case Report and Therapeutic Considerations

**DOI:** 10.7759/cureus.113122

**Published:** 2026-07-21

**Authors:** Patricia Flores Troche, Circe K. Ruiz Palafox, Agustín Zadkiel Cruz Torres, Astrid Dzoara Fuentes-Cruz, Sandra Estefanía Mejía Ruiz, Cristian Mejía Herrera, Ximena Gintare Alvarez Estrada

**Affiliations:** 1 Internal Medicine, Universidad Nacional Autónoma de México, Mexico City, MEX; 2 Dermatology, Hospital General de México Dr Eduardo Liceaga, Mexico City, MEX; 3 Internal Medicine, Universidad Autónoma de Querétaro, Querétaro, MEX; 4 General Medicine, Instituto Politécnico Nacional, Mexico City, MEX

**Keywords:** advanced melanoma, c-kit, immunotherapy, metastasis, mutation, targeted therapy

## Abstract

Melanoma is an aggressive malignancy characterized by significant molecular heterogeneity and poor prognosis in advanced stages. Activating *KIT* mutations occur predominantly in acral and mucosal melanoma subtypes and may represent actionable therapeutic targets. However, immunohistochemical expression of C-KIT (CD117) should not be interpreted as evidence of an activating *KIT* mutation, as molecular confirmation is required before targeted therapy can be considered. We report the case of a 39-year-old man with rapidly progressive metastatic melanoma involving the lungs, liver, and central nervous system. Histopathological examination demonstrated metastatic melanoma with positive C-KIT (CD117) immunohistochemical expression. Because of the patient's rapid clinical deterioration before referral for comprehensive molecular evaluation, molecular characterization could not be completed, precluding confirmation of activating *KIT* mutations and assessment for targeted therapy. This case highlights the importance of distinguishing immunohistochemical protein expression from actionable molecular alterations, emphasizes the value of timely molecular testing in advanced melanoma, and illustrates the practical challenges of implementing precision oncology in patients presenting with advanced-stage disease.

## Introduction

Melanoma is the most aggressive form of skin cancer because of its remarkable metastatic potential and poor prognosis once distant dissemination occurs. Although it accounts for only a small proportion of all cutaneous malignancies, its incidence continues to rise worldwide. According to the most recent Global Cancer Observatory (GLOBOCAN) estimates, 331,647 new cases and 58,645 deaths from cutaneous melanoma were reported in 2022 [1]. While the five-year survival rate exceeds 95% for localized disease, survival decreases dramatically in metastatic disease, underscoring the importance of early diagnosis and the identification of biomarkers with diagnostic, prognostic, and therapeutic value [2].

Melanoma is a molecularly heterogeneous disease characterized by recurrent alterations involving several oncogenic pathways, including mutations in BRAF, NRAS, and, less frequently, the KIT proto-oncogene [3]. Activating KIT mutations occur predominantly in acral, mucosal, and chronically sun-damaged melanomas and represent actionable molecular targets in selected patients [3]. However, it is important to distinguish activating KIT mutations from immunohistochemical expression of C-KIT (CD117). While immunohistochemistry detects receptor protein expression and serves as a valuable adjunct in the characterization of melanocytic lesions and the selection of patients for molecular testing, positive CD117 staining alone does not demonstrate the presence of an activating KIT mutation. Confirmation of such mutations requires dedicated molecular analysis before targeted therapy can be considered [3,4].

Beyond its role in identifying patients who may benefit from molecular testing, C-KIT immunohistochemical expression contributes to the differential diagnosis of challenging melanocytic lesions, particularly when distinguishing metastatic melanoma from other soft tissue neoplasms with melanocytic differentiation. Nevertheless, these findings should always be interpreted within the appropriate clinical, histopathological, and molecular context, since protein expression alone does not predict responsiveness to KIT-targeted therapy [5].

Current management of metastatic melanoma is primarily based on immune checkpoint inhibitors, whereas targeted therapies are reserved for tumors harboring actionable molecular alterations, including selected activating KIT mutations [2]. Despite remarkable advances in precision oncology, access to comprehensive molecular characterization remains limited in many healthcare settings, particularly in patients with rapidly progressive disease.

Here, we report the case of a patient with advanced metastatic melanoma showing immunohistochemical expression of C-KIT (CD117), in whom molecular characterization could not be completed because of rapid clinical deterioration before referral for specialized molecular evaluation. This case highlights the diagnostic value of immunohistochemistry, the importance of distinguishing protein expression from actionable genomic alterations, and the practical challenges of implementing precision oncology in patients presenting with advanced-stage melanoma.

## Case presentation

**Figure 1:**
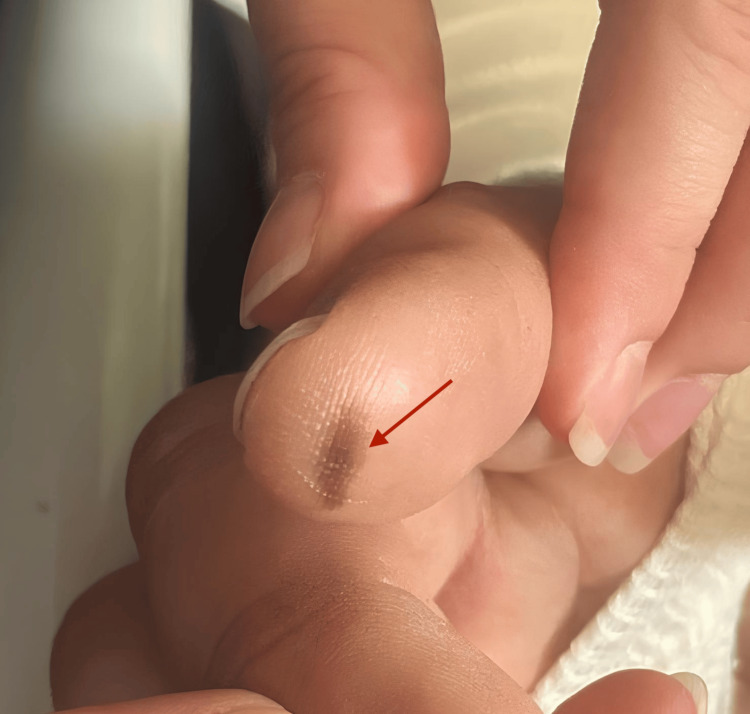
Pigmented lesion of the right index finger. Clinical photograph showing a dark-brown macular lesion located on the distal phalanx of the right index finger (red arrow). The lesion was not biopsied at the time of presentation; therefore, its role as the primary site of melanoma could not be established. Image resolution was enhanced using iLoveIMG through pixel upscaling for publication purposes only; no scientific content was altered (https://www.iloveimg.com).

**Figure 2:**
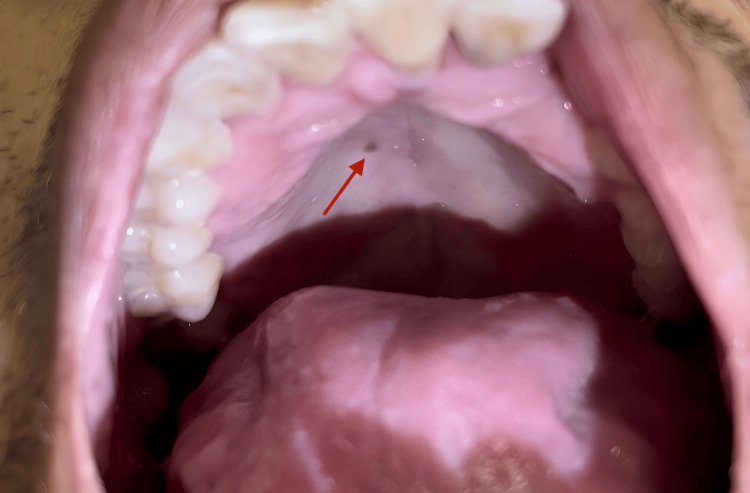
Pigmented lesion of the soft palate. Clinical photograph showing a hyperpigmented macule on the soft palate (red arrow). The lesion was not biopsied, and its relationship to the primary melanoma could not be definitively established. Image resolution was enhanced using iLoveIMG through pixel upscaling for publication purposes only; no scientific content was altered.(https://www.iloveimg.com).

A 39-year-old man, a driver residing in the State of Mexico, with no known comorbidities, presented with advanced metastatic melanoma. In September 2022, a dark-brown macular lesion measuring approximately 1.0 × 0.5 cm with irregular, poorly defined borders was identified on the right index finger (Figure 1). A second hyperpigmented macule measuring approximately 2 × 2 mm with similarly irregular and poorly defined borders was also observed on the soft palate (Figure 2). Neither lesion underwent further diagnostic evaluation or biopsy at that time. Consequently, the primary site of the melanoma could not be established with certainty, and the disease was considered compatible with acral melanoma, mucosal melanoma, or melanoma of unknown primary origin.

**Figure 3:**
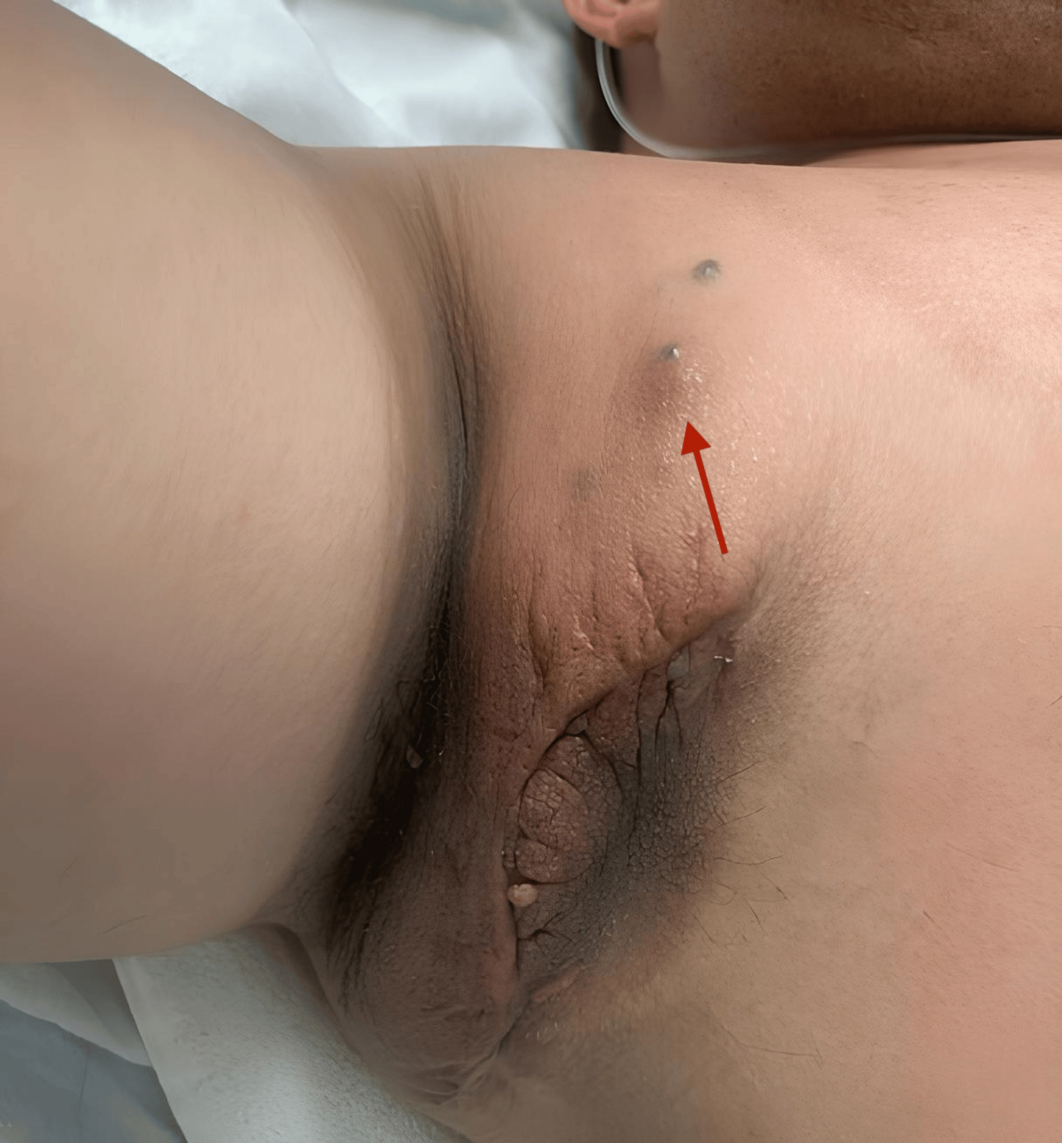
Pigmented nodules of the anterior upper trunk. Clinical photograph showing rapidly enlarging blue-black nodules on the ipsilateral anterior upper trunk (red arrow). These lesions were subsequently biopsied, and histopathological examination confirmed metastatic melanoma. Image resolution was enhanced using iLoveIMG through pixel upscaling for publication purposes only; no scientific content was altered.(https://www.iloveimg.com).

The current illness began in December 2024 with the development of a recurrent pustule-like lesion associated with progressive swelling of the right axillary region. Simultaneously, two rapidly enlarging, painless blue-black nodules measuring approximately 5 × 5 mm developed on the ipsilateral anterior upper trunk (Figure 3).

The patient was initially evaluated by a private physician, who requested staging studies. Contrast-enhanced computed tomography demonstrated a conglomerate of enlarged right axillary lymph nodes together with bilateral pulmonary nodules highly suggestive of metastatic disease. Axillary lymph node resection and biopsy of the associated axillary skin lesions were subsequently performed. Histopathological examination of these specimens confirmed metastatic melanoma.

The patient was later admitted to Hospital General Regional No. 200 Tecámac because of acute respiratory failure secondary to progressive pulmonary metastatic involvement. Contrast-enhanced whole-body computed tomography demonstrated widespread metastatic disease involving the lungs, liver, and central nervous system, including the cerebellum, pons, right temporal lobe, and left parietal lobe. According to the eighth edition of the American Joint Committee on Cancer (AJCC) staging system, these findings were consistent with stage IV melanoma (M1d), a category associated with a particularly poor prognosis because of the presence of brain metastases [6]. Baseline serum lactate dehydrogenase (LDH), an important prognostic biomarker in advanced melanoma, was not available in the patient's medical record at the time of staging (Table 1).

**Table 1:** Timeline of clinical course and diagnostic evaluation.

Date	Clinical Event
September 2022	Pigmented macular lesion identified on the right index finger and a hyperpigmented lesion observed on the soft palate; neither lesion underwent diagnostic evaluation or biopsy.
December 2024	A recurrent pustule-like lesion and progressive right axillary swelling developed. Simultaneously, rapidly enlarging blue-black nodules appeared on the ipsilateral anterior upper trunk.
Early 2025	Contrast-enhanced computed tomography demonstrated a conglomerate of enlarged right axillary lymph nodes together with bilateral pulmonary nodules highly suggestive of metastatic disease.
Early 2025	Axillary lymph node resection and biopsy of the associated axillary skin lesions were performed. Histopathological examination confirmed metastatic melanoma.
Hospital admission	The patient was admitted because of acute respiratory failure secondary to progressive pulmonary metastatic involvement.
Staging evaluation	Whole-body contrast-enhanced computed tomography demonstrated pulmonary, hepatic, and central nervous system metastases (cerebellum, pons, right temporal lobe, and left parietal lobe), consistent with stage IV (M1d) melanoma.
Histopathologic review	Axillary skin specimens demonstrated diffuse proliferation of atypical epithelioid melanocytes with a desmoplastic pattern, tumor necrosis, and abundant melanin pigment.
Immunohistochemistry	Tumor cells demonstrated positive C-KIT (CD117) expression by immunohistochemistry.
Oncologic management	The patient was referred for consideration of stereotactic radiosurgery and systemic palliative therapy; however, follow-up information was unavailable.

**Figure 4:**
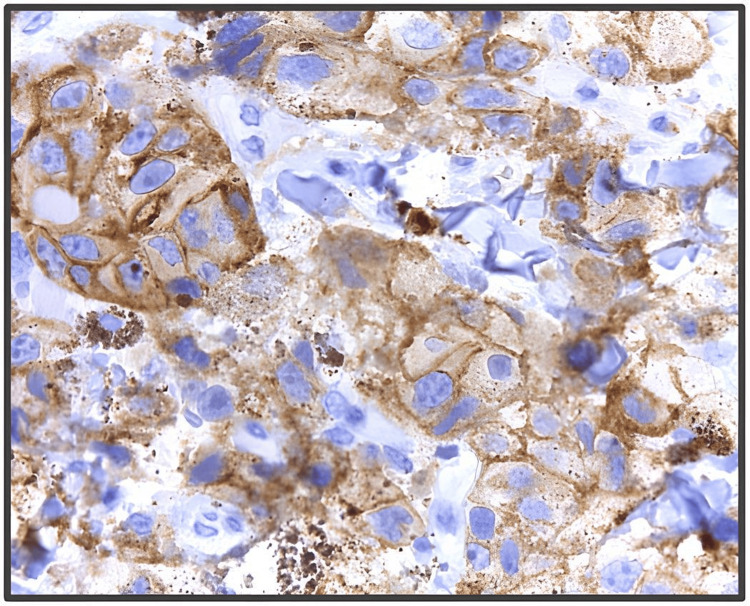
C-KIT (CD117) immunohistochemistry of the axillary skin specimen. Microphotograph of the axillary skin specimen demonstrating diffuse cytoplasmic and membranous C-KIT (CD117) expression (immunohistochemistry, original magnification ×400). Positive CD117 staining indicates immunohistochemical protein expression and should not be interpreted as evidence of an activating KIT mutation in the absence of molecular testing. Image resolution was enhanced using iLoveIMG through pixel upscaling for publication purposes only; no scientific content was altered.(https://www.iloveimg.com).

**Figure 5:**
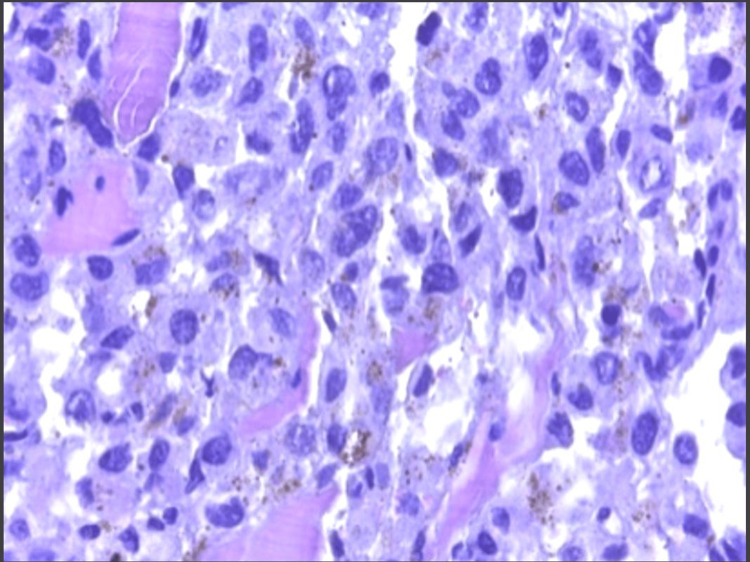
Histopathological examination of the axillary skin specimen. Hematoxylin and eosin stain (original magnification ×400) demonstrating atypical epithelioid melanocytes with nuclear pleomorphism, prominent nucleoli, and variable intracellular melanin pigment, consistent with metastatic melanoma. Image resolution was enhanced using iLoveIMG through pixel upscaling for publication purposes only; no scientific content was altered.(https://www.iloveimg.com).

**Figure 6:**
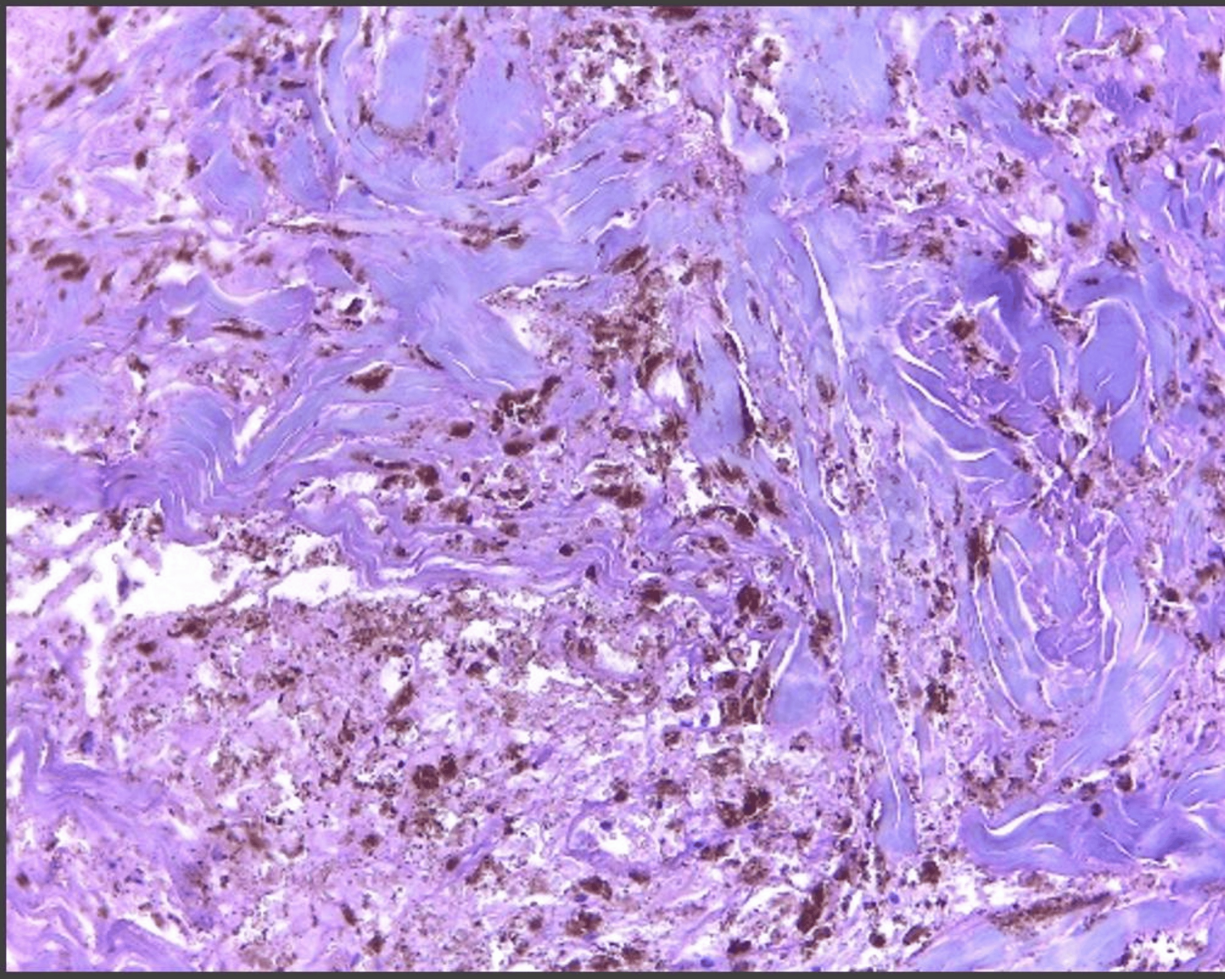
Histopathological examination of the axillary skin specimen. Hematoxylin and eosin stain (original magnification ×200) demonstrating dermal infiltration by atypical melanocytes associated with abundant intra- and extracellular melanin pigment. Image resolution was enhanced using iLoveIMG through pixel upscaling for publication purposes only; no scientific content was altered.(https://www.iloveimg.com).

A subsequent histopathological review of the axillary skin specimens demonstrated diffuse proliferation of atypical epithelioid melanocytes involving both the superficial and deep dermis, accompanied by extensive collagenized areas producing a desmoplastic pattern, foci of tumor necrosis, and abundant melanin pigment. These dermal infiltrates corresponded specifically to the axillary skin specimens and not to the original pigmented lesions of the index finger or soft palate. Immunohistochemical analysis demonstrated positive C-KIT (CD117) expression, representing receptor protein expression only rather than evidence of an activating KIT mutation (Figures 4-6). Because of the patient's rapidly progressive clinical deterioration, comprehensive molecular characterization could not be completed. Consequently, eligibility for molecularly targeted therapy based on KIT status could not be determined. Following confirmation of the diagnosis, the patient was evaluated by the Medical Oncology Department and referred to the National Medical Center Siglo XXI for consideration of stereotactic radiosurgery and systemic palliative therapy. 

 The patient was referred for palliative treatment; however, follow-up information was unavailable.

## Discussion

Advanced metastatic melanoma remains one of the most lethal malignancies despite the remarkable therapeutic advances achieved during the last decade. The present case illustrates several of the major challenges that continue to limit patient outcomes, including delayed diagnosis, uncertainty regarding the primary tumor site, rapidly progressive metastatic disease, and restricted access to comprehensive molecular characterization. The coexistence of untreated acral and mucosal pigmented lesions, neither of which underwent biopsy at the time of presentation, further complicated identification of the primary lesion and likely contributed to the advanced stage at diagnosis.

One of the most relevant aspects of this case is the interpretation of C-KIT (CD117) immunohistochemical positivity. Although C-KIT expression is more frequently observed in acral and mucosal melanomas, immunohistochemical positivity alone should not be interpreted as evidence of an activating KIT mutation or as a direct indication for KIT-targeted therapy. Current evidence supports molecular testing as the standard approach for identifying actionable KIT alterations, since only specific activating mutations have demonstrated clinically meaningful responses to tyrosine kinase inhibitors. In our patient, molecular characterization could not be completed because of the fulminant clinical course before referral for specialized molecular evaluation, illustrating a common challenge encountered in real-world practice, particularly in healthcare systems with limited access to advanced molecular diagnostics [3,4].

The therapeutic landscape of metastatic melanoma has changed substantially with the introduction of immune checkpoint inhibitors and molecularly targeted therapies. Long-term follow-up of the CheckMate 067 trial established nivolumab plus ipilimumab as an effective first-line strategy capable of providing durable survival benefits in selected patients with advanced melanoma. More recently, the RELATIVITY-047 trial demonstrated that nivolumab combined with relatlimab offers an additional first-line option with a more favorable toxicity profile. Nevertheless, these pivotal trials predominantly enrolled patients with good performance status and generally excluded individuals with uncontrolled or symptomatic brain metastases, limiting the direct applicability of their findings to patients with extensive metastatic disease such as the one presented here [7,8].

Similarly, tumor-infiltrating lymphocyte (TIL) therapy with lifileucel represents an important advance for patients with melanoma progressing after immune checkpoint inhibition [9]. However, this strategy requires adequate functional status, specialized infrastructure, and sufficient time for cell manufacturing, conditions that are frequently absent in patients with fulminant metastatic disease. Consequently, although therapeutic options have expanded considerably, many patients presenting with extensive tumor burden and rapid clinical deterioration remain ineligible for these highly specialized treatments.

The present case also underscores the importance of early recognition of pigmented lesions arising in acral and mucosal locations. Unlike conventional cutaneous melanoma, these subtypes often develop in anatomical sites that are not routinely examined and may initially resemble benign lesions, contributing to delayed diagnosis. In this patient, failure to investigate either of the original pigmented lesions prevented early histopathological confirmation and eliminated the opportunity for timely molecular characterization and therapeutic planning.

The principal contribution of this report is not the demonstration of an actionable KIT alteration, but rather the illustration of the limitations encountered when comprehensive molecular characterization cannot be completed in a patient with rapidly progressive metastatic melanoma. This case emphasizes the need to distinguish immunohistochemical C-KIT (CD117) expression from activating KIT mutations, reinforces the importance of timely molecular testing whenever feasible, and highlights the persistent gap between advances in precision oncology and their practical implementation in patients presenting with advanced-stage disease.

## Conclusions

This case describes an aggressive presentation of advanced metastatic melanoma characterized by rapid clinical progression, multiorgan dissemination, and central nervous system involvement. The presence of untreated pigmented lesions in both acral and mucosal locations, together with the absence of an identifiable primary lesion, illustrates the diagnostic challenges associated with these uncommon melanoma subtypes and emphasizes the consequences of delayed diagnosis.

An important contribution of this report is the distinction between immunohistochemical C-KIT (CD117) expression and activating KIT mutations. Although positive CD117 staining may support the characterization of melanocytic lesions and guide the selection of patients for molecular testing, it should not be interpreted as evidence of an actionable KIT mutation or as an indication for KIT-targeted therapy in the absence of molecular confirmation. This case also highlights the practical limitations of precision oncology in patients presenting with rapidly progressive metastatic disease. Despite the availability of highly effective systemic therapies for selected patients with advanced melanoma, delayed diagnosis, poor functional status, extensive tumor burden, and limited access to comprehensive molecular testing may preclude individualized treatment strategies and reduce the opportunity to benefit from targeted therapeutic approaches.

Ultimately, this report reinforces the importance of early recognition of acral and mucosal pigmented lesions, timely histopathological evaluation, and prompt molecular characterization whenever feasible. Earlier diagnosis, multidisciplinary management, and equitable access to molecular diagnostics remain essential to optimize therapeutic decision-making, particularly in patients with acral or mucosal melanoma, and ultimately improve outcomes for patients with advanced melanoma.
